# Assessing homing failure in honeybees exposed to pesticides: Guez's (2013) criticism illustrates pitfalls and challenges

**DOI:** 10.3389/fphys.2013.00352

**Published:** 2013-12-02

**Authors:** Mickaël Henry

**Affiliations:** ^1^INRA (Institut National de la Recherche Agronomique), UR406 Abeilles et EnvironnementAvignon, France; ^2^UMT Protection des Abeilles dans l'Environnement, Site AgroparcAvignon, France

**Keywords:** homing failure, honey bee, pesticide, sublethal dose, thiamethoxam

I have been involved in a publication showing that sublethal exposures to thiamethoxam, a neonicotinoid pesticide, increase the risk of homing failure in foraging honeybees (Henry et al., 2012a). Along with other recent toxicological studies on free-ranging bees (Gill et al., 2012; Schneider et al., 2012; Whitehorn et al., 2012), those results have motivated the European Food Safety Agency (EFSA, 2012) to reconsider the relevance of risk assessment of plant protection products on bees, currently based on the sole lethality criterion. Among the potential indicators of sublethal hazard, homing failure is a suitable candidate (EFSA, 2013) because (i) it integrates multiple physiological and cognitive functions such as orientation, spatial memory, associative learning and muscular flight activity, and (ii) it may be straightforwardly converted into a mortality rate. Homing failure studies are now being replicated for extending the study of behavioral impairments to other substances (Matsumoto, 2013) and even to pathogens (Li et al., 2013). The forthcoming expansion of homing studies in bees underscores the need to set standards for homing failure measurement. In our original contribution (Henry et al., 2012a), we calculated mortality due to homing failure, *m*_hf_, as the proportion of nonreturning *treated* foragers relative to expectations given by the proportion of returning *control* foragers. This *relative* control-treatment homing difference returns a mortality probability equivalent to the statistical *effect size* of the exposure.

However, Guez (2013a) criticized our calculation of *m*_hf_, arguing that dividing the homing difference by control expectations falsely inflates the mortality estimates. Instead, he claimed *m*_hf_ should simply read as the *absolute* control-treatment homing difference. Beside our in-depth reply, alerting on the necessity to properly fix experimental biases (Henry and Decourtye, 2013), Guez (2013b) persisted in his criticism. As a surrogate for our *relative* homing difference formula [Equation 1 in Guez (2013b)], he recommends either the use of his *absolute* homing difference formula [Equation 2 in Guez (2013b)] or an alternative formula measuring the proportional increase in post-exposure homing failure [Equation 3 in Guez (2013b)].

I show here that both alternatives are intractable and cannot be properly implemented in a honeybee population dynamics model (Khoury et al., 2011) as in our original study (Henry et al., 2012a). I aim to resolve the disagreement by clarifying several key features of the population models involved. I understand that Guez (2013a,b) implicitly assumes that homing experiments are based on the same temporal scale than the parameters of the honeybee population dynamics model (Khoury et al., 2011). This, however, is incorrect, as shown below. His tentative Equation 2 underestimates mortality, while Equation 3 may return severely overestimated mortalities. I hope this cautionary note will help risk assessors identifying some important pitfalls and challenges in the assessment of post-exposure homing failure in bees.

## A mortality probability can't be more than 100%

A mortality probability can't exceed 1 (i.e., 100% of the initially surveyed individuals died). Yet, Guez's (2013b) Equation 3 returns *m*_hf_ values of up to 155%. Readers should be aware that Equation 3 does actually not estimate a mortality rate *per se*, but a relative difference in homing failure. The resulting values are not bounded within [0,1] and therefore should *not* be combined with natural mortality as shown in subsequent equations. As control homing failure gets closer to 0, *m*_hf_ in Equation 3 (Guez, 2013b) rapidly gets much larger than 1 and eventually tends to ∞. So does the result of the subsequent equation, which is intended to give daily mortality rate for use in the colony population model. Guez's (2013b) Equation 3 is therefore inappropriate and liable to overestimate mortality. The hypothesis of a low or null control homing failure is plausible and is even a desirable property for any homing experiment. For instance, control homing failure was only 1.5% in experiment 3 of Henry et al. (2012a). In Guez's (2013b) example, if one substitutes the 17% control homing failure by a 1.5% value, Equation 3 would return *m*_hf_ = (0.432 − 0.015)/0.015 = 27.8. Subsequently, total daily mortality would reach an unrealistic level of *m*_total_ = 0.154 + 0.154 × 27.8 = 4.44 (foragers have an overall daily mortality probability of 444%).

## A mortality probability is dimensionless

By definition, probabilities and proportions are dimensionless. Yet, Guez (2013b) recurrently expresses homing failure in *individuals.day*^−1^, and even insists on this property to justify the combination of different mortality parameters (*“Importantly, since [Control homing success], [Control homing failure], [Treatment homing success], and [Treatment homing failure] are expressed in* individuals.day^−1^, *m*_hf_ is also expressed in individuals.day^−1^.*”*). This is however incorrect. Homing success (or failure) is a ratio between numbers of individuals (e.g., [nb of individuals failing homing]/[nb of individuals initially surveyed]), and therefore is dimensionless. The same holds true for mortality probabilities, which are ratios between numbers of individuals that have died and numbers of initially alive individuals (see also the dimensionless values in Khoury et al., 2011). I acknowledge that a daily mortality value was erroneously expressed in *individuals.day*^−1^ at one point in our original study (Henry et al., 2012a). Unfortunately, the subsequent critical reasoning was based on this false assumption (Guez, 2013a,b), promoting a spurious use of mortality parameters.

## Natural mortality occurring during the homing experiment is not a daily natural mortality

In conjunction with the improper use of the *individuals.day*^−1^ unit, the sources of homing failure were erroneously assigned a daily basis [Equations 4 and 5 in Guez (2013b)]. However, the homing experiment is *not* fixed over time. Most of the surveyed honeybees had completed their homing flight at 30 min. of release only and others several hours later. The *daily* basis in the homing experiment approach is provided by the pesticide dose, which is intended to reproduce the daily pesticide residue intake by honeybees foraging on a treated crop. Some bees failed homing because they naturally died in the course of the experiment, but this natural mortality may not be assigned to a determined period of time. This is among others why the simple control-treatment homing differences [Equation 2 in Guez (2013b)] may not be combined with the *daily* mortality probability in population dynamics models, unless it is beforehand corrected by an appropriate reference value (Henry and Decourtye, 2013). From a statistical standpoint, this is equivalent to calculating an *effect size*. The tentative Equation 3 in Guez (2013b) actually incorporates such a correction, but does not eventually return a mortality rate *sensu stricto*.

## Homing failure due to experimental stress in not trivial

Beyond the temporal mismatch, it is critical to properly account for any bias resulting from the experimental stress. Guez's Equation 2 seems to hold true if “ *[… ] we assume that most of the homing failure observed in the control is due to natural predation*” (Guez, 2013b). This assumption is however too far reaching because the experimental stress substantially contributes to homing failure in *Control* bees. One might tentatively estimate the magnitude of experimental stress in *Control* bees by comparing homing statistics from the most challenging experiment *vs*. from the least challenging one [i.e., Experiments 2 and 3, respectively, in Henry et al. (2012a)]. When released from an unfamiliar place 1 km away from their colony, 16.9% of the control bees failed homing. When released from a familiar place and in the vicinity of their colony, only 1.5% of the bees failed homing. The 15.4% difference is therefore mostly attributable to the increased experimental challenge imposed to bees, and is of the same order of magnitude as homing failure attributable to pesticide exposure (though the most challenging experiment might also include a greater natural mortality simply due to increased homing duration). This needs be investigated in further studies.

## Future challenges in homing failure assessment

I have highlighted here some pitfalls on the way to assess honeybee homing failure in the context of dietary exposure to pesticides. Most importantly, homing experiments can't be assigned the same temporal basis as the daily population parameters in colony dynamics models (Khoury et al., 2011), and this must be properly accounted for.

Important challenges for future studies are listed below.

- Homing failure and (sub-)chronicle exposure. As pointed out by Guez (2013a) and also in earlier discussions (Cresswell and Thompson, 2012; Henry et al., 2012b), homing failure was evidenced when the entire daily intake of a forager is consumed in a single dose (acute exposure). In real exposure events, the intake is fractioned over several foraging bouts (sub-chronicle exposure). Repeated sub-chronicle exposures have been successfully carried out in small-scale experimental setups (Schneider et al., 2012) but are to date technically difficult to monitor at home range scales, *i.e*. with homing distances encompassing kilometers.
- Combining homing failure *m*_hf_ with daily natural mortality. Implementing mortality due to homing failure into population dynamics models might be further improved. In earlier studies (Cresswell and Thompson, 2012; Henry et al., 2012a), *m*_hf_ was combined with daily natural mortality *m*_natural_ as an additional independent probability (*m*_total_ = m_natural_ + m_hf_). One might however consider that post-exposure homing failure only occurs at the end of the foraging day, and therefore only applies on the portion of foragers who escaped natural mortality that day. Under this assumption, total mortality would be slightly reduced (*m*_total_ = m_natural_ + m_hf_ − [m_natural_ × m_hf_]). I suggest to search into that direction to set a lower bound scenario for *m*_hf_.
- Disentangling the contributions of natural mortality and experimental stress in homing experiments. As shown earlier, homing failure due simply to the experimental condition is arguably not trivial. One last challenge is the assessment of the relative contribution of natural mortality and experimental conditions to total homing failure, i.e., the magnitude of the experimental effect that must be controlled for in the calculation of *m*_hf_.
